# Anthocyanins Promote Learning through Modulation of Synaptic Plasticity Related Proteins in an Animal Model of Ageing

**DOI:** 10.3390/antiox10081235

**Published:** 2021-07-31

**Authors:** David Vauzour, Catarina Rendeiro, Alfonsina D’Amato, Pierre Waffo-Téguo, Tristan Richard, Jean Michel Mérillon, Matthew G. Pontifex, Emily Connell, Michael Müller, Laurie T. Butler, Claire M. Williams, Jeremy P. E. Spencer

**Affiliations:** 1 Faculty of Medicine and Health Sciences, Norwich Medical School, University of East Anglia, Norwich NR4 7UQ, UK; M.Pontifex@uea.ac.uk (M.G.P.); E.Connell@uea.ac.uk (E.C.); Michael.Muller@uea.ac.uk (M.M.); 2 School of Sport, Exercise and Rehabilitation Sciences, University of Birmingham, Edgbaston, Birmingham B15 2TT, UK; c.rendeiro@bham.ac.uk; 3 Department of Pharmaceutical Sciences, University of Milan, 20133 Milan, Italy; alfonsina.damato@unimi.it; 4 UFR des Sciences Pharmaceutiques, Unité de Recherche Œnologie EA 4577, University of Bordeaux, USC 1366 INRA, Equipe Molécules d’Intérêt Biologique, 210 Chemin de Leysotte, F-33882 Villenave d’Ornon, France; pierre.waffo-teguo@u-bordeaux.fr (P.W.-T.); tristan.richard@u-bordeaux.fr (T.R.) ; jean-michel.merillon@u-bordeaux.fr (J.M.M.); 5 Faculty of Science and Engineering, Anglia Ruskin University, Cambridge CB1 1PT, UK; Laurie.butler@aru.ac.uk; 6 School of Psychology and Clinical Language Sciences, University of Reading, Reading RG6 6AL, UK; claire.williams@reading.ac.uk; 7 Molecular Nutrition Group, Department of Food and Nutritional Sciences, School of Chemistry, Food and Pharmacy, University of Reading, Reading RG6 6AP, UK; j.p.e.spencer@reading.ac.uk

**Keywords:** flavonoids, brain, signalling, cognition, neuroprotection

## Abstract

Anthocyanin-rich foods, such as berries, reportedly ameliorate age-related cognitive deficits in both animals and humans. Despite this, investigation into the mechanisms which underpin anthocyanin-mediated learning and memory benefits remains relatively limited. The present study investigates the effects of anthocyanin intake on a spatial working memory paradigm, assessed via the cross-maze apparatus, and relates behavioural test performance to underlying molecular mechanisms. Six-week supplementation with pure anthocyanins (2% *w*/*w*), administered throughout the learning phase of the task, improved both spatial and psychomotor performances in aged rats. Behavioural outputs were accompanied by changes in the expression profile of key proteins integral to synaptic function/maintenance, with upregulation of dystrophin, protein kinase B (PKB/Akt) and tyrosine hydroxylase, and downregulation of apoptotic proteins B-cell lymphoma-extra-large (Bcl-xL) and the phosphorylated rapidly accelerated fibrosarcoma (p-Raf). Separate immunoblot analysis supported these observations, indicating increased activation of extracellular signal-related kinase (ERK1), Akt Ser473, mammalian target of rapamycin (mTOR) Ser2448, activity-regulated cytoskeleton-associated protein (Arc/Arg 3.1) and brain-derived neurotrophic factor (BDNF) in response to anthocyanin treatment, whilst α-E-catenin, c-Jun N-terminal kinase (JNK1) and p38 protein levels decreased. Together, these findings suggest that purified anthocyanin consumption enhances spatial learning and motor coordination in aged animals and can be attributed to the modulation of key synaptic proteins, which support integrity and maintenance of synaptic function.

## 1. Introduction

A significant proportion of neurodegenerative conditions, as well as more generalised cognitive decline, can be attributed to modifiable lifestyle factors such as diet [1]. Diet has been increasingly acknowledged for its impact on brain health and strongly influences age-related cognitive decline [2], as well as neurodegenerative disease incidence and onset [3]. Diet therefore represents an important means that can be used to support both healthy ageing and the attenuation of neurological disease risk. Phytochemical constituents such as flavonoids have emerged over the past decades as key influencers of brain health and disease and are being avidly investigated in order to determine their neuroprotective potential [4,5], boosted by their apparent multi-target actions. Epidemiological studies highlight this potential, with increased total flavonoid consumption positively associated with improved cognitive and memory function amongst adults [6,7], slower rates of cognitive decline amongst the elderly [8], and reduced risk of neurodegenerative diseases, such as Parkinson’s [9] and Alzheimer’s diseases [10,11].

A subgroup of flavonoids known as anthocyanins, water-soluble plant pigments particularly abundant in berries, have been recognised for their bioactive properties and are known to influence numerous disease processes. These effects appear to extend to the brain, with anthocyanins gaining attention for their protective effects against age-related neurodegeneration and cognitive decline [12,13]. Consistent with these observations, preclinical studies indicate that anthocyanins improve learning and memory across several mammalian species [13,14], with anthocyanin-rich blueberry intake shown to induce spatial memory improvements in young animals [15,16] and ameliorate age-related cognitive decline in old animals [17,18,19,20]. Current human studies are in agreement with pre-clinical research findings. Indeed, increased blueberry and strawberry anthocyanin consumption [8,21,22,23,24,25] is associated with improved cognitive functions amongst young healthy adults [26], whilst cognitive decline and Alzheimer’s dementia risk is reduced amongst elderly non-demented adults. Furthermore, dietary intervention studies in humans utilising anthocyanin-rich plant or food extracts indicate an ability of these dietary components to improve cognitive performance in older adults [27,28] and in patients with mild cognitive impairment [29]. Mechanistically, enhancement of neuronal signalling and synaptic plasticity [16,30], neuronal resilience [31], glucose metabolism/insulin resistance [32,33] and modulation of the microbiota and its subsequent functions [34] have been forwarded as plausible modes of action; however, further elucidation is required.

Anthocyanins have considerable potential in regard to brain health and disease. We previously reported that supplementation with pure anthocyanins (2% *w*/*w*) for 6 weeks resulted in an enhancement of spatial working memory in 18-month-old rats. These behavioural changes coincided with a regional increase of brain derived neurotrophic factor (BDNF) mRNA expression within the hippocampus, highlighting a potential mechanism of action attributable to the memory improvements observed in these aged animals [20]. Here, we extend upon this report by further elucidating the underlying mechanisms of actions associated with anthocyanin-rich extract, specifically neuronal signalling and synaptic function/integrity. In contrast to previous investigations in which feeding of animals occurs during the post-task training period, the present study specifically focusses on a novel cognitive paradigm aiming at understanding how supplementation with anthocyanin-rich extract during the cross-maze apparatus task may enhance the learning capabilities of aged naïve animals (e.g., supplementation is concomitant to task learning in the cross-maze apparatus).

## 2. Materials and Methods

### 2.1. Study Approval

Experimental procedures and protocols were reviewed and approved by the Animal Welfare and Ethical Review Body (AWERB) and were conducted within the provisions of the Home Office Animals (Scientific Procedures) Act 1986 (ethical protocol code: 30/4246). Utmost effort was utilised to prevent suffering and minimize the numbers of animals required for these experiments.

### 2.2. Extraction and Analysis of the Anthocyanins

The anthocyanin fraction was prepared by extracting anthocyanins from blueberries (*Vaccinium corymbosum*) giving rise to a pre-purified extract. Briefly, 5 kg of frozen blueberries harvested in SCEA Dittmeyer Agricola (Domaine des Myrtilles “le Auqueyres”, Parentis en Born, France) were first ground and extracted for 2 h under constant stirring in 2.5 L of methanol-trifluoroacetic acid (999:1) at room temperature. After filtration on glass wool, the residue was extracted a second time as described above and filtrated. The filtrates were combined and concentrated to 1 L under reduced pressure at 35 °C. The residual layer was then extracted with (6 × 1 L) of methyl tert-butyl ether (MtBE). The MtBE extract was discarded and the aqueous extract containing anthocyanins was freeze-dried. Enhancement of its anthocyanins content was achieved by adsorption on Amberlite XAD-7 washed by H_2_O-trifluoroacetic acid (999:1), followed by elution with methanol-trifluoroacetic acid (999:1); this provided 16.3 g of a pre-purified extract. The identification of anthocyanins was achieved by a combination of liquid chromatography electrospray ionisation tandem mass spectrometry (LC-ESI-MS/MS) and liquid chromatography tandem nuclear magnetic resonance (LC-NMR) analyses as described previously [35]. Liquid Chromatography tandem mass spectrometry (LC MS/MS) analyses were carried out on a chromatography apparatus, Agilent 1200 from Agilent Technologies (Santa Clara, CA, USA) coupled to an Esquire 3000+ ion trap mass spectrometer using an electrospray ionisation (ESI) source from Bruker Daltonics (Billerica, MA, USA). For LC-NMR analyses, the chromatography apparatus was similar to that of LC–MS/MS and was coupled to a Bruker AVANCE III 600 MHz spectrometer equipped with a ^1^H–^13^C inverse detection flow probe from Bruker BioSpin (Rheinstetten, Germany) (Table S1). Anthocyanin content was estimated at 62.4% enrichment from calibration curves using appropriate analytical methods [36].

### 2.3. Animals and Dietary Supplementation

Sixteen male Wistar rats aged 16 months at the beginning of the experiments were purchased from Harlan (Blackthorn, UK). Animals were maintained in a controlled environment (21 ± 2 °C), humidity (55 ± 10%), a standard light-dark cycle (12 h/12 h) and fed ad libitum on a standard chow diet (RM3-P, Special Diet Services, Essex, UK). Following 2 weeks of acclimatisation, animals were then pseudo-randomly assigned to 2 groups (*n* = 8 per group, starting body weight: 512 ± 37 g) based on their baseline spatial working memory correct choice scores and matched across all eight animals per group.

Pure anthocyanin extracts obtained as described above were incorporated (2% *w*/*w*) into the standard AIN-76A purified diet for rodents (Research Diets, New Brunswick, NJ, USA) and made into dry pellets for animal consumption. Both interventions used were analytically well characterised, isocaloric and macro- and micronutrient-matched (Table S2). Body weight and food intake were recorded twice a week. Immediately after acclimatization, naïve rats were fed either the anthocyanin diet or the control for a duration of 6 weeks. Spatial working memory was assessed at the beginning and end of the feeding period as described below.

Following the 6-week intervention, an overdose of sodium pentobarbital (150 mg/Kg, i.p) was administered. Blood was drawn via cardiac puncture and centrifugated (10 min, 1300× *g*, room temperature) to obtain sera, whilst ice-cold saline (100 mL) containing 10 U/mL of sodium heparin was transcardially perfused prior to isolation of brains. All samples were snap-frozen in liquid nitrogen and stored at −80 °C until further analysis.

### 2.4. Spatial Working Memory Testing

#### 2.4.1. Habituation and Shaping Sessions

Rats were tested in a cross-maze apparatus as described previously [17,20]. During the course of the habituation period the rats were maintained in a food-deprived state. Prior to experimental testing and supplementation, animals were subjected to 6 weeks of shaping sessions (2 × shaping sessions per week and 6 × trials per session), to ensure animals could reliably obtain rewards from maze arms. In each trial animals were required to enter an open goal arm and collect a reward pellet from the food well (entry to the al-ternate goal arm was restricted). The ‘open’ goal arm was varied between trials according to a pseudorandom schedule (Supplementary Figure S1).

#### 2.4.2. Alternation Task

Immediately after habituation (baseline) and completion of the 6-week shaping sessions, test sessions were conducted. A pseudo-random sequence of correct arm choices, as well as the starting arm choice, was selected for each test session. Rats (unfasted state) were subjected to testing sessions as previously described [17]. Briefly, animals received 8 trials per test session with a 5 min intertrial interval, with each trial consisting of a sample and choice phase (ref previous paper). Upon completion of a trial the animal was returned to its cage for 5 min before commencing the next trial. Accuracy and latency to make a choice were measured for each trial. Ethanol solution with a 50% concentration was used to clean the maze after each trial to remove olfactory cues.

### 2.5. Accelerating Rotarod

Fine motor coordination, balance, and resistance to fatigue were quantitated by measuring the amount of time an animal could remain standing on a rotating, accelerating Rota Rod (Harvard Apparatus, Cambridge, Cambridgeshire, UK). The rod consists of a drum, 60 mm in diameter, which could be varied for rotational speed. Each rat was individually placed upon the rod which was initially set at 2 rpms. Once the animal maintained its grip/orientation without assistance, the rod steadily accelerated in increments for five minutes (2 rpms per 30 s) until 20 rpms was reached. Time spent upon the apparatus (maximum = 300 s) was recorded.

### 2.6. Antibody Microarray

The Panorama Antibody Microarray Cell Signalling Kit (Sigma-Aldrich, Amersham, UK) was used as described previously [37]. Statistical analysis was conducted on biological replicates and significantly modulated protein expression in the hippocampus is reported in Supplementary Table S4.

### 2.7. Western Immunoblotting

Dissected brain regions were homogenized on ice with a glass homogenizer (10 strokes) using Tris (50 mM), Triton X-100 (0.1%), NaCl (150 mM) and EGTA/EDTA (2 mM); pH 7.4, containing mammalian protease inhibitor cocktail (1:100 dilution), sodium pyrophosphate (1 mM), phenylmethylsulfonyl fluoride (10 µg/mL), sodium vanadate (1 mM) and sodium fluoride (50 mM). Homogenates were left on ice for 45 min before centrifugation at 1000× *g* for 5 min at 4 °C to remove unbroken cell debris and nuclei. Protein concentration in the supernatants was determined by the Pierce BCA assay (Thermofisher, Loughborough, UK). For analysis of proteins by Western immunoblotting, samples were incubated for 5 min at 95 °C in boiling buffer (final conc. 62.5 mM Tris, pH 6.8, 2% sodium dodecyl sulfate, 5% 2-mercaptoethanol, 10% glycerol and 0.0025% bromophenol blue).

The processed samples (20–40 µg/lane) were loaded and run on a 9–12% SDS-polyacrylamide gel and after separation transferred to nitrocellulose membranes (Hybond-ECL; Sigma-Aldrich, Amersham, UK) by semi-dry electroblotting (1.5 mA/cm^2^). The nitrocellulose membrane was then blocked (20 mM Tris, pH 7.5, 150 mM NaCl; Tris-buffered saline (TBS) containing 4% (*w*/*v*) skimmed milk powder) for 45 min at room temperature and washed twice for 5 min in TBS supplemented with 0.05% (*v*/*v*) Tween 20 (TTBS). Blots were then incubated with either anti-phospho-p44/42 MAPK (Erk1/2) (Thr 202/Tyr 204) pAb (1:1000 dilution), anti ERK1/ERK2 pAb (1:1000), anti-phospho-SAPK/JNK (Thr 183/Tyr 185) pAb (1:1000), anti-SAPK/JNK (1:1000) pAb, anti-phospho-p38 MAPK (Thr 180/Tyr 182) pAb (1:1000), anti p38 MAPK pAb (1:1000), anti-phospho-Akt (Ser 473) pAb (1:1000), anti-Akt pAb (1:1000), anti-phospho-mTOR (Ser 2448) pAb (1:1000), anti-mTOR pAb (1:1000), anti-Arc/Arg3.1 pAb (1:1000), anti-BDNF pAb (1:1000), anti-α-E-catenin pAb (1:500) or anti-glyceraldehyde 3-phosphate dehydrogenase (GAPDH) pAb (1:5000), in TTBS containing 1% (*w*/*v*) skimmed milk powder (antibody buffer) overnight at room temperature on a three dimensional rocking table. After this, blots were washed twice for 10 min in TTBS and incubated with goat anti-rabbit IgG conjugated to HRP (1:1000 dilution) for 60 min. Blots were given a final wash twice for 10 min in TTBS rinsed in TBS and covered in enhanced chemiluminescence reagent for 1–2 min. Images were developed and band intensities were analysed using Image J software (version 1.52p, National Institutes of Health, Bethesda, MD, USA) [38]. Molecular weight markers were used for band identification (MW 27,000–180,000 and MW 6500–45,000 (Sigma Aldrich, Amersham, UK) and were run in parallel with the samples. Equal loading and efficient transfer were confirmed through Ponceau Red staining.

### 2.8. Statistical Analysis

All behavioural data were subjected to two-way analysis of variance for repeated measures with group (placebo, anthocyanins) and time (0, 6 weeks) as main factors. The *p* values were corrected for multiple testing by Benjamini–Hochberg correction to control for false discovery rate. For the protein array data evaluation, statistics and differentially regulated protein identification were carried out using the Perseus software v1.6.1.3 (http://www.perseus-framework.org (accessed on 18 December 2019); Max Planck Institute of Biochemistry, Martinsried, Planegg, Germany). The protein log2-fold changes were evaluated by using the comparison of absorbance between the control and treated experimental groups. The two samples test was applied, with the following criteria: student’s *t*-test, S0 value of 0 on both sides, permutation-based false discovery rate (FDR) value of 0.05. Gene Ontology analyses and overexpressed test (Fisher’s exact, *p* value < 0.05 and FDR < 0.1), related to *Rattus norvegicus* reference database, were performed by Panther Classification System (www.pantherdb.org, accessed on 18 December 2019). For the Western immunoblotting data, the statistical evaluation of the results was performed by unpaired *t*-test. All statistical analyses unless otherwise stated were conducted using GraphPad Prism version 8 (GraphPad Software, San Diego, CA, USA).

## 3. Results

### 3.1. Weight and Food Intake

A significant increase in body weight was observed over the time course of the experiment (F (1, 16) = 10.74; *p* = 0.0047) and was similar across both experimental groups (control: 25.1 ± 18.3 g; anthocyanin: 27.9 ± 18.0 g), with no significant effect of treatment established (F (1, 16) = 0.4604; *p* = 0.5071). In addition, there was no significant change in food intake established for either of the two dietary groups (*p* > 0.05), with control and anthocyanin group consuming on average 28.2 g and 30.6 g food/day, respectively. Average anthocyanin consumption was determined to be 6.12 mg/day/rat.

### 3.2. Shaping Sessions

**Figure 1:**
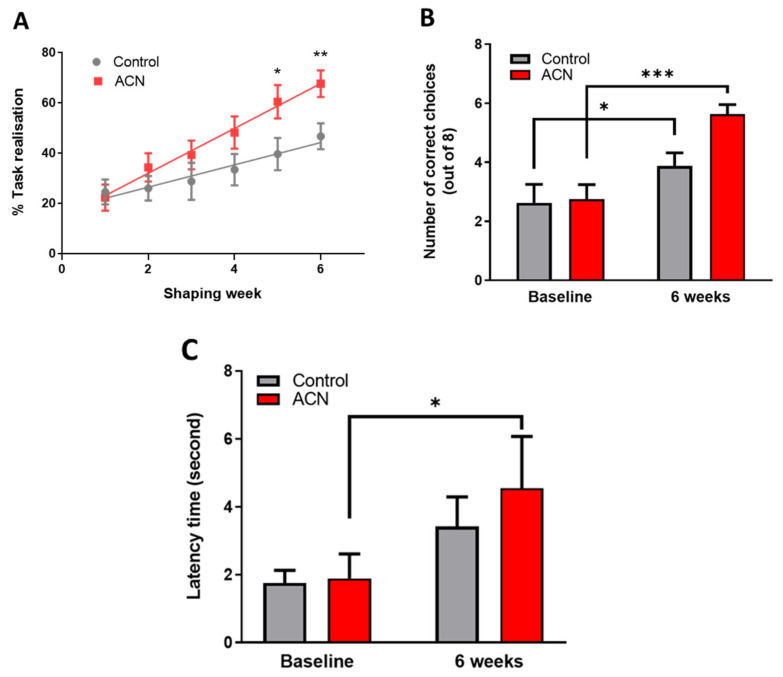
(**A**) Effect of anthocyanin (ACN) supplementation on learning the cross-maze rules during the shaping sessions. Results are presented as percentage ± SEM of task realisation (*n* = 8). Two-way ANOVA indicated a significant time (F (5, 153) = 12.80, *p* < 0.0001) and treatment (F (1, 34) = 4.892, *p* = 0.0338) but no significant interaction (F (5, 153) = 1.584, *p* = 0.1677) impact on task realisation. Bonferroni post-hoc test indicated a greater effect of anthocyanins on completing the task versus control treatment following 6 weeks of shaping sessions. * *p* < 0.05, ** *p* < 0.01, *** *p* < 0.001. (**B**) Effect of anthocyanin (ACN) supplementation on spatial memory performance measured as choice accuracy (number of correct choices) in a T maze apparatus. Maximum score is eight correct choices. Results are presented as means ± SEM (*n* = 8). Two-way ANOVA indicated a significant time effect (F (1, 14) = 39.09, *p* < 0.0001) and interaction (F (1, 14) = 6.067, *p* = 0.0273), but no significant effect of treatment F (1, 14) = 2.465, *p* = 0.1387) on overall cognitive performance. Bonferroni post-hoc test analysis indicated a greater effect of the anthocyanin-rich extract versus control on spatial working memory following 6 weeks of shaping sessions. (**C**) Effect of anthocyanin (ACN) supplementation on latency time to fall from the rotating rod. Results are presented as means ± SEM (*n* = 8). The latency time to fall was significantly affected by time (F (1, 14) = 14.72, *p* = 0.0024) but not by treatment (F (1, 12) = 0.2566, *p* = 0.6217). No interaction between treatment and time was observed (F (1, 12) = 0.7910, *p* = 0.3913). Subsequent post-hoc analysis revealed that anthocyanin fed animals maintained a better dynamic equilibrium (*p* = 0.0017) compared to those fed a control diet (*p* = 0.1183) following 6 weeks of supplementation.

Immediately after the 2-week habituation period, a test session aiming to assess the animals’ spatial working memory was started. As anticipated, untrained naive animals performed below chance (e.g., 4 out of 8 trials) at baseline with the number of scores reaching on average 2.7 ± 1.5 correct choices. Animals were split into two balanced groups according to baseline performance, forming group 1 (2.6 ± 1.8 correct choices) which were allocated placebo and group 2 (2.8 ± 1.4 correct choices) which were allocated the anthocyanin treatment (*p* > 0.05). Thereafter and whilst being fed either the control or anthocyanin-rich extract, animals were habituated to the rules of the cross-maze through successive training sessions denominated thereafter shaping sessions for a period of 6 weeks. There was a highly significant effect of time (F (5, 153) = 12.80, *p* < 0.0001) and treatment (F (1, 34) = 4.892, *p* = 0.0338), but no significant interaction (F (5, 153) = 1.584, *p* = 0.1677) over the 6-week period indicating that the animals learned the rules of the task, with anthocyanin-rich extract fed animals performing better than the control fed animals. Post-hoc analysis indicated a trend towards significant improvements in task realisation (in relation to control) by the fourth week of supplementation with the anthocyanin-rich extract (*p* = 0.0544), with the effect maintained throughout the remainder of the intervention period (week 5, *p* < 0.05 and week 6, *p* < 0.01) (Figure 1A).

### 3.3. Spatial Working Memory

Six weeks of shaping sessions in the cross-maze apparatus had a significant impact on working spatial memory. Indeed, a highly significant effect of time (F (1, 14) = 39.09, *p* < 0.0001) and interaction (F (1, 14) = 6.067, *p* = 0.0273), but no significant effect of treatment F (1, 14) = 2.465, *p* = 0.1387) was observed on the animals’ overall cognitive performance. As anticipated, control animals significantly improved by 1.5 ± 0.5-fold their working spatial memory following 6-week shaping sessions (*p* < 0.05), indicating they had learnt/acquired the rules of the cross-maze apparatus. However, feeding anthocyanin-rich extract for 6 weeks resulted in an improvement of 2.1 ± 0.3-fold (*p* < 0.001) of the overall cognitive performance of the animals in the alternation task of the spatial working memory test (Figure 1B). Animals receiving the anthocyanin-rich extract performed on average 0.7 ± 0.5-fold better than the control fed animals (*p* = 0.00024) at 6 weeks. Time taken to complete the task was also measured in addition to the choice accuracy, revealing no significant differences among groups during the acquisition of the task (not shown).

### 3.4. Motor Skills

As a measure of general motor skills, the dynamic equilibrium of the animals was tested on a rotating rod, and data are presented as latency time to fall. The latency to fall was significantly affected by time (F (1, 14) = 14.72, *p* = 0.0024) but not by treatment (F (1, 12) = 0.2566, *p* = 0.6217), nor by the time and treatment interaction (F (1, 12) = 0.7910, *p* = 0.3913). Subsequent post-hoc analysis revealed that treatment with anthocyanin-rich extract improved dynamic equilibrium (*p* = 0.0017) compared with placebo control (*p* = 0.1183) following 6 weeks of supplementation (Figure 1C). No impact of time nor treatment was observed for the rotating speed of the rod (not shown).

### 3.5. Hippocampal Protein Expression

**Figure 2:**
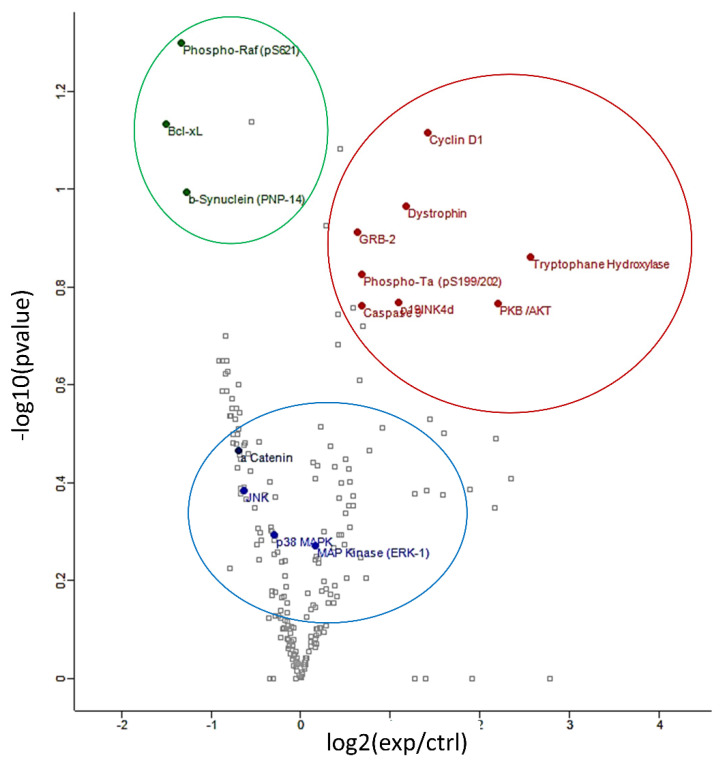
Volcano plot of differentially regulated proteins identified by protein array: under-expressed proteins are shown in green, overexpressed proteins are shown in red and proteins verified by Western blot analyses are shown in blue.

An antibody microarray was employed in these experiments as it represents a powerful tool for examining the abundance of numerous large proteins simultaneously. The expression profile of hippocampal proteins involved in signal transduction, neuroplasticity, apoptosis and cell cycle regulation was found to be significantly modulated (*p* < 0.05) following 6 weeks’ intake of anthocyanin-rich extract compared to the iso-caloric control. The protein array analyses resulted in more than 200 quantified proteins (Supplementary Table S1). Figure 2 depicts a volcano plot of proteins quantified by the protein array approach. The overexpressed proteins are highlighted in red, on the right of graph. The under-expressed proteins are showed in green, on the left of the graph. 

**Figure 3:**
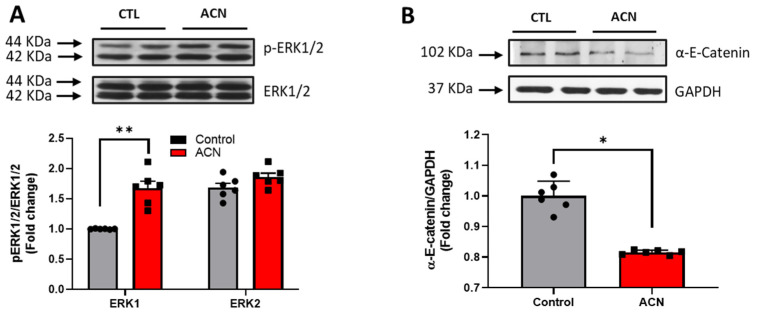

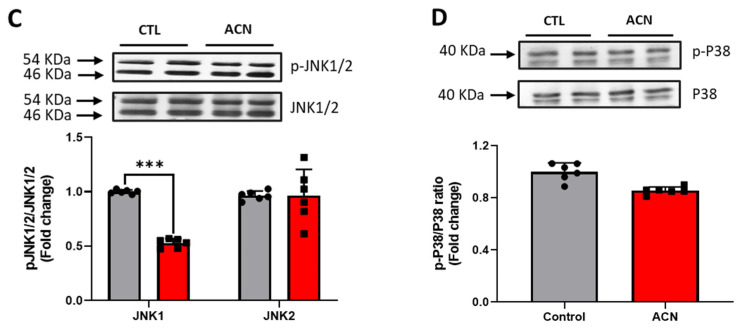
Immunoblotting analysis of proteins extracted from the hippocampus. Crude lysates (20–40 µg protein) were immunoblotted with antibodies that specifically recognise (**A**) phospho-ERK1/2, total ERK1/2; (**B**) α-E-catenin and GAPDH; (**C**) phospho-JNK1/2, total JNK1/2 and (**D**) phospho-p38, total-p38. Results (phospho/total ratio for ERK1/2, JNK1/2, and p38; protein/GAPDH ratio for α-E-catenin) are presented as means ± SEM (*n* = 6). Band intensities were determined by densitometric analysis using Fiji Image J. *** *p* < 0.001; ** *p* < 0.01; * *p* < 0.05: indicate significant differences versus control. CTL = control; ACN = anthocyanins.

Proteins of particular interest identified in the protein array were confirmed via Western Blot analyses (blue circle in the graph). ERK1 protein was 1.5–2-fold increased (*p* < 0.01) in the anthocyanin treated group whilst ERK2 proteins were unaffected (Figure 3A). In contrast to ERK proteins, α-E-catenin (*p* < 0.05), JNK1 (*p* < 0.001) and p38 (*p* > 0.05, ns) protein levels were down regulated following anthocyanin intake (Figure 3B–D).

**Figure 4:**
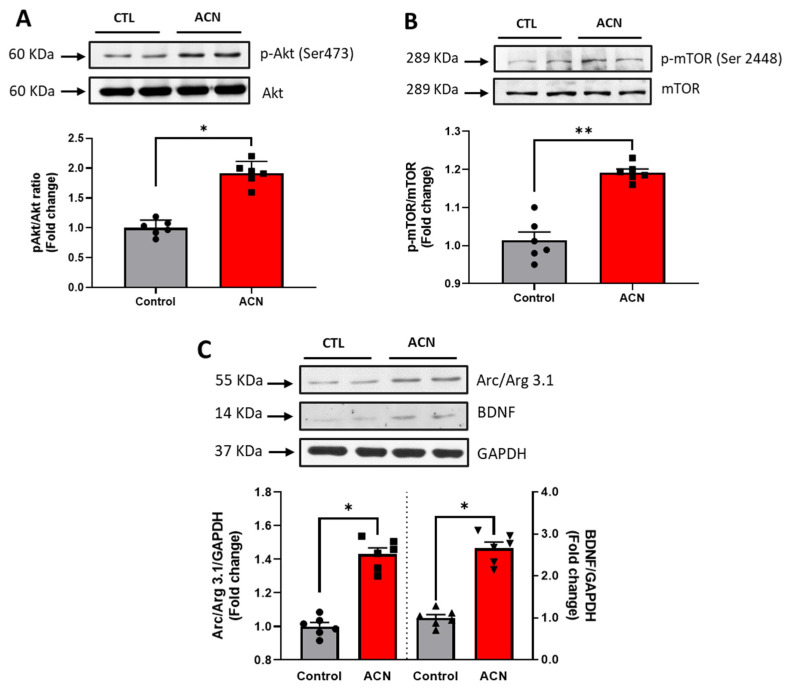
Immunoblotting analysis of proteins extracted from the hippocampus. Crude lysates (20–40 µg protein) were immunoblotted with antibodies that specifically recognise (**A**) phospho-Akt Ser 473, total Akt; (**B**) phospho mTOR Ser 2448, total mTOR amd (**C**) Arc/Arg 3.1, BDNF and GAPDH. Results (phospho/total ratio for Akt Ser473, mTOR Ser 2448; protein/GAPDH ratio for Arc/Arg3.1 and BDNF) are presented as means ± SEM (*n* = 6). Band intensities were determined by densitometric analysis using Fiji Image J. ** *p* < 0.01; * *p* < 0.05: indicate significant differences versus control. CTL = control; ACN = anthocyanins.

Probing further proteins involved in synaptic plasticity maintenance, we identified Akt Ser 473 (*p* < 0.05), mTOR Ser 2448 (*p* < 0.01), Arc/Arg 3.1 (*p* < 0.05) and BDNF (*p* < 0.05) to be positively modulated by the anthocyanin-rich extract treatment (Figure 4A–C).

**Figure 5:**
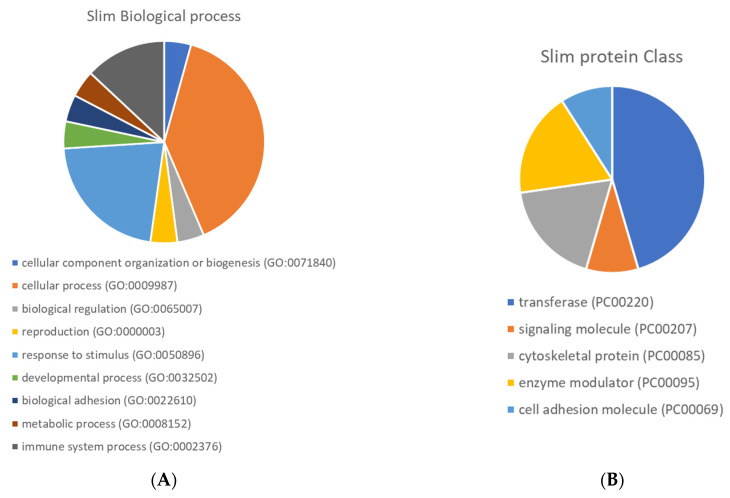
Pie chart of Gene Ontology enrichment of biological process (**A**) and protein class (**B**) terms.

Figure 5 presents the Gene Ontology enrichment of protein class and biological process terms. The differentially expressed proteins were enriched in several protein classes, such as transferase, cell adhesion molecule and signalling molecule. Overexpression analyses were performed using the Panther Gene Ontology Overexpression Test (Pathway) and the results are presented in Supplementary Table S5. The main enriched pathways were: interferon-gamma signalling pathway, the Janus kinase (JAK)-signal transducer and activator of transcription (STAT) signalling pathway, Toll-like receptor signalling pathway, Alzheimer’s disease-amyloid secretase pathway, rat sarcoma virus (RAS) pathway, B cell activation, Gastrin and cholecystokinin receptors (CCKR) signalling map, oxidative stress response.

## 4. Discussion

Anthocyanin-rich foods (specifically blueberries and strawberries) have been shown to be effective at reversing age-related deficits in spatial memory and can enhance different aspects of synaptic plasticity [19,39,40], a process severely affected by ageing [41,42]. For example, a 2% (*w*/*w*) blueberry diet significantly improved spatial working memory through activation of the ERK-CREB-BDNF pathway, an important modulatory pathway of synaptic plasticity [17]. Furthermore, supplementation with pure anthocyanins for 6 weeks at levels similar to that found in blueberry (2% *w*/*w*) resulted in an enhancement of spatial memory in 18-month-old rats [20]. The improvement in spatial memory induced by the anthocyanin was equal to that induced by whole blueberry, suggesting anthocyanins are principally responsible for efficacy of the whole fruit in vivo. Whilst most studies to date have focussed on memory consolidation and retrieval in ageing models, the present study investigated the impact of chronic consumption of anthocyanin extract on the learning phase of the spatial working memory paradigm, and therefore assessed learning capacity of 16-months old rats. Our results demonstrate that over the course of the 6-week shaping phase, animals receiving the anthocyanin extract performed better in the cross-maze apparatus, indicating an enhancement of learning capabilities. In addition, performance in the cross-maze apparatus was independent of the improvement of motor skills, indicating a clear cognitive impact of the anthocyanin treatment.

The enhancement of spatial memory performance induced by the anthocyanin-rich extract was supported by subsequent molecular analysis, which revealed a significant modulation of hippocampal protein expression that likely underpins these behavioural effects. These proteins are involved in metabolic, immune and cell adhesion molecule processes. Previously we have observed that BDNF, a major regulator of synaptic transmission and plasticity at adult synapses [43], is activated through the ERK-CREB-BDNF pathway and may mediate the influence of anthocyanin-rich foods on spatial memory [16,17,20]. In agreement with these results, we again observed a significant modulation of ERK1/2 and BDNF by the anthocyanin-rich extract treatment. The concomitant increase in BDNF observed in our anthocyanin-rich diet-supplemented aged animals may be responsible for the enhancements in spatial working memory. Such improvement may be mediated in part through the activation of the PI3K/Akt pathway, the activation of mTOR through the phosphorylation at Ser 2448, along with the subsequent neurotransmitter release in the synaptic cleft [44]. In corroboration with these proposed mechanisms, increased phosphorylation of both Akt Ser 473 and its downstream target mTOR Ser 2448 was established in the aged anthocyanin treated animals. Further downstream, hippocampal protein levels of activity-regulated cytoskeleton-associated protein Arc/Arg3.1, which has been associated with memory improvements and long-term potentiation (LTP), mirrored the level of both BDNF and ERK1/2. Such findings therefore suggest that the increase in Arc/Arg3.1 may be under the regulatory control of both BDNF and ERK1/2 in the hippocampus.

Anthocyanin-rich extract treatment upregulated the expression of a range of cytoskeletal proteins in the hippocampus, including dystrophin, plakoglobin, vinculin, microtubule associated protein 1 (MAP1b) and spectrin. Such proteins have integral roles at neuronal membranes where they support learning and memory processes [45] and regulate synaptogenesis [46]. For example, lack of dystrophin in the hippocampus has been reported to affect cognitive functions [47], spatial memory and long-term potentiation [48]. In addition to cytoskeleton proteins, anthocyanin intake significantly affected the expression of the pro-apoptotic caspase-3 and anti-apoptotic proteins, such as Bcl-xL and other members of the Bcl-2 family. Although Bcl-xL has been reportedly implicated in Alzheimer’s disease [49], recent evidence highlights a role for this protein in neurite outgrowth [50,51], synaptic plasticity [52,53], and mitochondrial bioenergetics [54,55]. Such reductions in Bcl-xL are therefore likely to promote cell survival [56]. In addition, while proteolytic caspases and in particular caspase-3 have been implicated in neurodegenerative disorders and Alzheimer’s disease [57], evidence also suggests that caspase-3 may play alternative roles which include modulation of synaptic plasticity along with learning and memory processes [58,59]. Such activation of caspase-3 following anthocyanin intake may therefore be related to improvement in learning and memory, although further work would be necessary to validate this hypothesis.

We previously reported similar findings in age-related memory deficit rodents fed a hydroxybenzoic acid and hydroxycinnamate-rich diet [37]. Such small phenolics may cross the blood–brain barrier via amino acid transporters [60] and can therefore act directly on protein signalling. Interestingly, following uptake anthocyanins are extensively metabolized by bacterial fermentation and absorption from the colon [61], giving rise to many degradation products including phenolic, hippuric, phenylacetic, and phenylpropenoic acids. Alternatively, anthocyanins may also affect the microbiota composition and function [62], which can then interact with brain function through the gut–brain axis [63].

## 5. Conclusions

This study indicates that the consumption of anthocyanin-rich extract improves learning capabilities in aged rats, counteracting spatial memory loss in aged brains, through the modulation of a number of cell signalling events implicated predominantly in synaptic plasticity, apoptosis and cytoskeleton remodelling. These processes act synergistically to maintain the number and strength of synaptic connections in the brain, an essential factor for efficient memory and cognitive functions.

## Data Availability

Data is contained within the article and Supplementary Materials.

## Supplementary Materials

The following are available online at https://www.mdpi.com/article/10.3390/antiox10081235/s1, Figure S1: Experimental design, Table S1: Raw data protein array results, Table S2: Chromatographic data (peak number and retention time) and MS2 *m*/*z* values (molecular and fragment ions) along with 1H-NMR chemical shifts of the anthocyanins detected., Table S3: Composition of the animal diets, Table S4: Proteins significantly (FDR adjusted *p* < 0.05) modulated by the anthocyanins treatment as assessed by the Protein microarray technique, Table S5: Panther gene Ontology Overexpression Test (Pathway).
