# Supplementary material for: Anthocyanins Promote Learning through Modulation of Synaptic Plasticity Related Proteins in an Animal Model of Ageing
Source: Antioxidants (Basel). 2021 Jul 31;10(8):1235. doi: 10.3390/antiox10081235 (PMC8388918; doi:10.3390/antiox10081235)

Supplementary information

Anthocyanins promote learning through modulation of synaptic plasticity related proteins in an animal model of ageing

David Vauzour [1,] *, Catarina Rendeiro [2], Alfonsina D'Amato [3], Pierre Waffo-Téguo [4], Tristan Richard [4], Jean Michel Mérillon [4], Matthew G. Pontifex [1], Emily Connell [1], Michael Müller [1], Laurie T Butler [5], Claire M. Williams [6] and Jeremy P.E. Spencer[7]

1. Norwich Medical School, Faculty of Medicine and Health Sciences, University of East Anglia, Norwich NR4 7UQ, United Kingdom; D.Vauzour@uea.ac.uk (DV); M.Pontifex@uea.ac.uk (MP); E.Connell@uea.ac.uk (EC), Michael.Muller@uea.ac.uk (MM)
2. School of Sport, Exercise and Rehabilitation Sciences, University of Birmingham, Edgbaston, Birmingham, B15 2TT, United Kingdom; c.rendeiro@bham.ac.uk (CR)
3. University of Milan, Department of Pharmaceutical Sciences, Milan, Italy; alfonsina.damato@unimi.it
4. Univ. Bordeaux, UFR des Sciences Pharmaceutiques, Unité de Recherche Œnologie EA 4577, USC 1366 INRA, Equipe Molécules d'Intérêt Biologique, 210 chemin de Leysotte, F-33882 Villenave d'Ornon, France; pierre.waffo-teguo@u-bordeaux.fr (PWT) ; tristan.richard@u-bordeaux.fr (TR) ; jean-michel.merillon@u-bordeaux.fr (JMM)
5. Faculty of Science and Engineering, Anglia Ruskin University, Cambridge, United Kingdom; Laurie.butler@aru.ac.uk (LTB)
6. School of Psychology and Clinical Language Sciences, University of Reading, Reading, RG6 6AL, United Kingdom; claire.williams@reading.ac.uk (CMW)
7. Molecular Nutrition Group, Department of Food and Nutritional Sciences, School of Chemistry, Food and Pharmacy, University of Reading, RG6 6AP, United Kingdom; j.p.e.spencer@reading.ac.uk (JPES)

*Correspondence: D.Vauzour@uea.ac.uk ; Tel.: +44-1603-591-732

Table S2: Chromatographic data (peak number and retention time) and MS2 m/z values (molecular and fragment ions) along with ^{1}H-NMR chemical shifts of the anthocyanins detected.

Peak no.	RT (min)	Molecular (*m/z*)	Fragment (*m/z*)	^{1}H-NMR spectrum	Compound
1	32.6	465	303	9.03 (1H, *s*, H-4) , 7.82 (2H, *s*, H-2′, H-6′) , 7.08 (1H, *s*, H-8) , 6.79 (1H, *s*, H-6) , 5.36 (1H, *d*, *J* = 7.7 Hz, H-1′′) , 4.09 (1H, *m*, H-2′′) , 3.99 (1H, *m*, H-4′′) , 3.86-3.80 (3H, H-6A′′, H-6B′′, H-3′′)	delphinidin-3-galactoside
2	33.7	465	303	9.03 (1H, *s*, H-4) , 7.82 (2H, *s*, H-2′, H-6′) , 7.09 (1H, *s*, H-8) , 6.79 (1H, *s*, H-6) , 5.45 (1H, *d*, *J* = 7.8 Hz, H-1′′) , 3.99 (1H, *dd*, *J* = 11.4 Hz, *J* = 1.4 Hz, H-6A′′) , 3.86 (1H, *m*, H-6B′′) , 3.75 (1H, *m*, H-2′′) , 3.70 (1H, *m*, H-3′′) , 3.65 (1H, *m*, H-5′′) , 3.54 (1H, *m*, H-4′′)	delphinidin-3-glucoside
3	34.8	449	287		cyanidin-3-glycoside
4	35.5	435	303	9.00 (1H, *s*, H-4) , 7.85 (2H, *s*, H-2′, H-6′) , 7.09 (1H, *s*, H-8) , 6.79 (1H, *s*, H-6) , 5.35 (1H, *d*, *J* = 7.3 Hz, H-1′′) , 4.14-4.09 (3H, H-2′′, H-4′′, H-5A′′) , 3.97-3.89 (2H, H-3′′, H-5B′′)	delphinidin-3-arabinoside
5	37.0	479	317	9.08 (1H, *s*, H-4) , 7.95 (1H, *s*, H-2′) , 7.89 (1H, *s*, H-6′) , 7.11 (1H, *s*, H-8) , 6.80 (1H, *s*, H-6) , 5.41 (1H, *d*, *J* = 7.8 Hz, H-1′′) , 4.09 (1H, *m*, H-2′′) , 4.06 (3H, *s*, OMe) , 3.99 (1H, *m*, H-4′′) , 3.86-3.80 (3H, H-6A′′, H-6B′′, H-3′′)	petunidin-3-galactoside
6	38.3	479	317	9.06 (1H, *s*, H-4) , 7.93 (1H, *s*, H-2′) , 7.89 (1H, *s*, H-6′) , 7.12 (1H, *s*, H-8) , 6.81 (1H, *s*, H-6) , 5.48 (1H, *d*, *J* = 7.8 Hz, H-1′′) , 4.06 (3H, *s*, OMe) , 4.00 (1H, *dd*, *J* = 11.4 Hz, *J* = 1.4 Hz, H-6A′′) , 3.91 (1H, *m*, H-6B′′) , 3.77-	petunidin-3-glucoside

				3.70 (2H, H-2'', H-3'') , 3.66 (1H, *m*, H-5'') , 3.55 (1H, *m*, H-4'')	
7	40.3	449	317	9.05 (1H, *s*, H-4) , 8.03 (1H, *s*, H-2') , 7.93 (1H, *s*, H-6'), 7.14 (1H, *s*, H-8) , 6.82 (1H, *s*, H-6) , 5.38 (1H, *d*, *J* = 7.4 Hz, H-1'') , 4.14-4.10 (3H, H-2'', H-4'', H-5A'') , 4.07 (3H, *s*, OMe) , 3.93-3.85 (2H, H-3'', H-5B'')	petunidine-3-arabinoside
8	41.5	493	331	9.12 (1H, *s*, H-4) , 8.03 (1H, *s*, H-2', H-6') , 7.14 (1H, *s*, H-8) , 6.83 (1H, *s*, H-6) , 5.40 (1H, *d*, *J* = 7.8 Hz, H 1'') , 4.09 (6H, *s*, OMe) , 4.06 (1H, *m*, H-2'') , 4.01 (1H, *m*, H-4'') , 3.85-3.80 (3H, H-6A'', H-6B'', H-3'')	malvidin-3-galactoside
9	43.1	493	331	9.12 (1H, *s*, H-4) , 8.03 (1H, *s*, H-2', H-6') , 7.17 (1H, *s*, H-8) , 6.85 (1H, *s*, H-6) , 5.52 (1H, *d*, *J* = 7.7 Hz, H 1'') , 4.10 (6H, *s*, OMe) , 4.00 (1H, *dd*, *J* = 11.4 Hz, *J* = 1.4 Hz, H-6A'') , 3.85 (1H, *m*, H-6B'') , 3.82-3.75 (2H, H-2'', H-3'') , 3.72 (1H, *m*, H-5'') , 3.58 (1H, *m*, H-4'')	malvidin-3-glucoside
10	45.5	463	331	9.09 (1H, *s*, H-4) , 8.06 (1H, *s*, H-2', H-6') , 7.19 (1H, *s*, H-8) , 6.86 (1H, *s*, H-6) , 5.38 (1H, *d*, *J* = 7.7 Hz, H 1'') , 4.14-4.10 (3H, H-2'', H-4'', H-5A'') , 4.11 (6H, *s*, OMe) , 3.98-3.90 (2H, H-3'', H-5B'')	malvidin-3-arabinoside

Table S3. Composition of the animal diets.

	Control diet (CTL)		Anthocyanin diet (ACN)	
**Macronutrients**	g / 100 g	kcal%	g / 100 g	kcal%
Protein	19	20	19	20
Carbohydrate	68	70	68	70
Fat	4	10	4	10
Total		100		100
kcal/g	3.85		3.85	
**Ingredient**	**g/kg**		**g/kg**	
Casein	200		200	
L-cysteine	3		3	
Corn starch	540		540	
Maltodextrin	35		35	
Sucrose	125		125	
Cellulose	50		50	
Soybean oil	45		45	
Mineral mix[1]	10		10	
Calcium[2]	19		19	
Potassium Citrate	17		17	
Vitamin mix[3]	10		10	
Anthocyanin extract	0		0.2	

[1] Mineral mix S10026 supplied in g/kg of premix triturated in sucrose: NaCl, 259; MgO, 41.9; $MgSO_4{\cdot}7H_2O$, 257.6; $(NH_4)2MoO_4{\cdot}4H_2O$, 0.3; $CrK(SO_4)_2 \cdot 12H_2O$, 1.925; $CuCO_3$, 1.05; $C_6H_5FeO_7$, 21; $MnCO_3 \cdot xH2O$, 12.25; KI, 0.035; NaF, 0.2; Na_2SeO_3, 0.035; $[ZnCO_3]_2$, 5.6.

[2] Combination of dicalcium phosphate ($CaHPO_4$) and calcium carbonate ($CaCO_3$)

[3] AIN-76A vitamin mix supplied in g/kg of premix triturated in sucrose: vitamin A acetate (500,000 IU/gm), 0.8; vitamin D3 (100,000 IU/gm), 1; vitamin E acetate (500 IU/gm), 10; menadione sodium bisulfite, 0.08; biotin (1%), 2; cyanocobalamin (0.1%), 1; folic acid, 0.2; nicotinic acid, 3; calcium pantothenate, 1.5; pyridoxine-HCl, 0.7; riboflavin, 0.6; thiamine-HCl, 0.6.

Table S4: Proteins significantly (FDR adjusted p<0.05) modulated by the anthocyanins treatment as assessed by the Protein microarray technique.

Sigma ID	*UniProt accession no.*	*Gene name*	*Protein name*	*SIGMA_Ab*	*SIGMA_Area*	*log2(exp/ctrl)*
Protein array						
T0678	P09810	*Tph1*	Tryptophane Hydroxylase	Tryptophane Hydroxylase Clone:WH-3	Neurobiology	2.56
P1601	P47196	*Akt1*	PKB/AKT	PKB/AKT	Signal Transduction	2.20
C7464	P39948	*Ccnd1*	Cyclin D1	Cyclin D1 Clone: DCS-6	Cell Cycle	1.42
D8043	P11530	*Dmd*	Dystrophin	Dystrophin Clone: MANDRA-1	Neurobiology	1.17
P4354	Q6P4Z7	*Cdkn2d*	Cdkn2d	p19INK4d Clone: DCS-100	Cell Cycle	1.10
C4356	Q9JHK1	*Casp9*	Caspase 9	Caspase 9 Clone: CAS9	Apoptosis	0.69
T6819	P19332	*Mapt*	Phospho-Tau (pS199/202)	Phospho-Tau (pS199/202)	Neurobiology	0.68
G2791	P62994	*Grb2*	GRB-2	GRB-2 Clone: GRB-232	Signal Transduction	0.63
S1436	Q63754	*Sncb*	b-Synuclein (PNP-14)	b-Synuclein (PNP-14)	Neurobiology	-1.27
R1151	Q80VX4	*Zhx2*	Phospho-Raf (pS621)	Phospho-Raf (pS621)	Signal Transduction	-1.33
B9429	P53563	*Bcl2l1*	Bcl-xL	Bcl-xL Clone: 2H12	Apoptosis	-1.50
Verified by Western Blot						
C2081	Q5U302	*Ctnna1*	a Catenin	a Catenin	Cytoskeleton	-0.69
J4500	P49185	*Mapk8*	JNK	JNK	Signal Transduction	-0.63
M0800	P63086	*Mapk1*	p38 MAPK	p38 MAPK	Signal Transduction	-0.30
M7927	P21708	*Mapk3*	MAP Kinase (ERK-1)	MAP Kinase RK-1)	Signal Transduction	0.16

Table S5: Panther gene Ontology Overexpression Test (Pathway)

PANTHER Pathway	*Rattus norvegicus (REF)*	#	*Expected*	*Fold Enrichment*	+/-	*P value*
Interferon-gamma signalling pathway	32	3	0.01	> 100	+	1.36E-05
JAK/STAT signalling pathway	17	1	0.01	> 100	+	7.99E-01
Toll receptor signalling pathway	64	3	0.02	> 100	+	1.08E-04
Alzheimer disease-amyloid secretase pathway	69	3	0.02	> 100	+	1.36E-04
Ras Pathway	80	3	0.02	> 100	+	2.11E-04
B cell activation	80	3	0.02	> 100	+	2.11E-04
CCKR signalling map	176	6	0.05	> 100	+	1.83E-10
Oxidative stress response	59	2	0.02	> 100	+	2.05E-02
TGF-beta signalling pathway	105	3	0.03	97.07	+	4.76E-04
Parkinson disease	110	3	0.03	92.65	+	5.47E-04
VEGF signalling pathway	76	2	0.02	89.4	+	3.40E-02

Figure S1: Experimental design

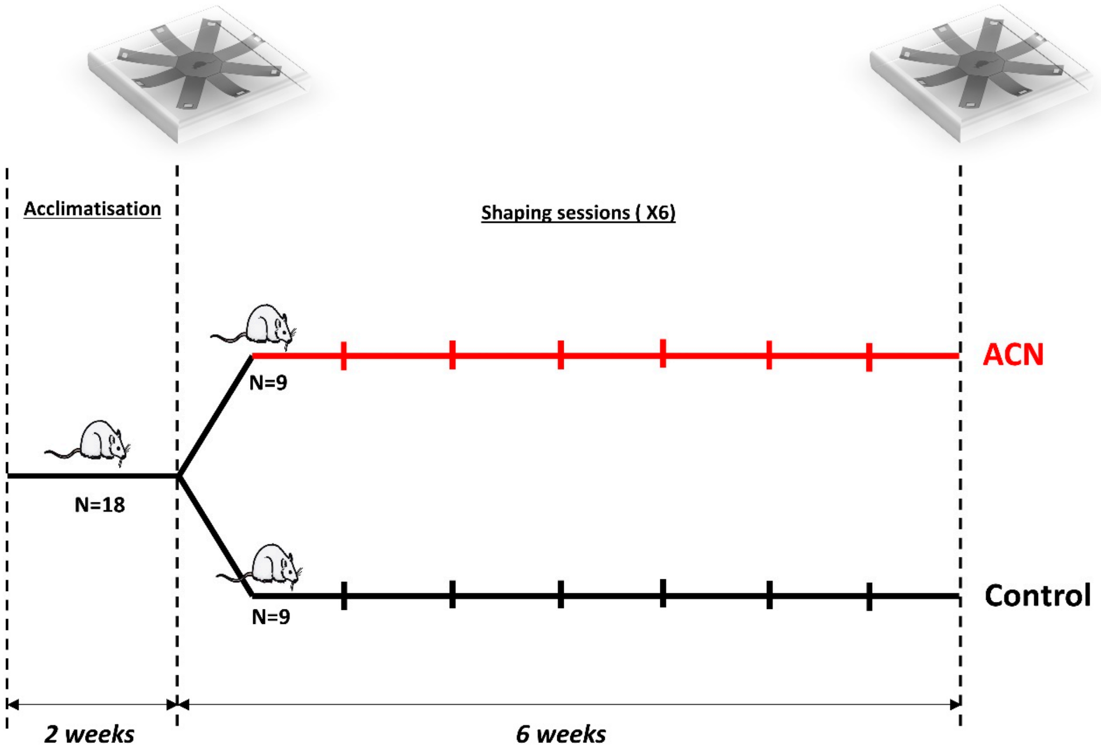

Supplement: Supplementary file 1 [file antioxidants-10-01235-s001.zip › Supplementary Tables and Figure Vauzour et al. Final.pdf]
